# Trends in the Development of Diagnostic Tools for Red Blood Cell-Related Diseases and Anemias

**DOI:** 10.3389/fphys.2020.00387

**Published:** 2020-05-26

**Authors:** Lars Kaestner, Paola Bianchi

**Affiliations:** ^1^Theoretical Medicine and Biosciences, Medical Faculty, Saarland University, Homburg, Germany; ^2^Experimental Physics, Faculty of Natural Science and Technology, Saarland University, Saarbrücken, Germany; ^3^Fondazione IRCCS Ca’ Granda Ospedale Maggiore Policlinico Milano, UOC Ematologia, UOS Fisiopatologia delle Anemie, Milan, Italy

**Keywords:** point of care, functional screening, physico-chemical properties, mobile laboratory, sickle cell disease, personalized medication, artificial intelligence

## Abstract

In the recent years, the progress in genetic analysis and next-generation sequencing technologies have opened up exciting landscapes for diagnosis and study of molecular mechanisms, allowing the determination of a particular mutation for individual patients suffering from hereditary red blood cell-related diseases or anemia. However, the huge amount of data obtained makes the interpretation of the results and the identification of the pathogenetic variant responsible for the diseases sometime difficult. Moreover, there is increasing evidence that the same mutation can result in varying cellular properties and different symptoms of the disease. Even for the same patient, the phenotypic expression of the disorder can change over time. Therefore, on top of genetic analysis, there is a further request for functional tests that allow to confirm the pathogenicity of a molecular variant, possibly to predict prognosis and complications (e.g., vaso-occlusive pain crises or other thrombotic events) and, in the best case, to enable personalized theranostics (drug and/or dose) according to the disease state and progression. The mini-review will reflect recent and future directions in the development of diagnostic tools for red blood cell-related diseases and anemias. This includes point of care devices, new incarnations of well-known principles addressing physico-chemical properties, and interactions of red blood cells as well as high-tech screening equipment and mobile laboratories.

## Do We Need Novel Diagnostic Tools?

There is a demand for novel diagnostic assays and devices from several perspectives. (i) Since we are still facing huge economic differences across our planet, there is a need (and a market) for low-cost diagnosis of common and rare red blood cell-related diseases. This includes sickle cell disease, thalassemia, malaria, and other less common hereditary and acquired hemolytic anemias. (ii) Scientific progress and the omics era allowed to unravel new diseases. This covers so far undiagnosed red blood cell diseases as well as identifying new pathogenetic variants in previously phenomenologically defined diseases. However, the huge amount of data obtained need to be interpreted, and often variants predicted at *in silico* analysis as possibly pathogenic—the so-called variants of unknown significance (VUS)—may have no or very little impact on protein function and obviously require further diagnostic tests to assess their functional involvement in the disease. (iii) Even knowing the molecular defect in diseases does not tell us much about the severity and the current state of the disease (e.g., severity of anemia, vaso-occlusive crisis in sickle cell disease). Thus, there is a need to establish the prognosis and possible complications of particular disease states and to determine an appropriate treatment. This is not restricted to the selection or combination of particular drugs but also the dose of these drugs. The concept of personalized theranostics addresses this issue but requires new approaches to become effective.

Given the rarity and the heterogeneity of this group of disorders, the interest of pharmaceutical companies and device maufacturers in developing drugs and technological devices, respectively was limited in the past. The strong possibility not to reach a sufficient volume of requests, the approach to rare disorders, in particular the congenital ones, was scanty up to some years ago. Funding initiatives for rare diseases, especially by the European Commission (within the 7th Framework Program and Horizon 2020) boosted research in the field of rare anemias, and this will continue within the coming years (Horizon Europe). A consensus document highlighting major achievements in diagnosis and treatment of blood disorders, including rare red blood cell disorders, and identifying the greatest unmet clinical and scientific needs has been recently prepared by more than 300 experts belonging to the European Hematology Association (EHA) (Engert et al., 2016).

## Point of Care Diagnostic Devices

There are many requirements that should be taken into consideration in the development of a point of care device. It is important that the test performed will be rapid, user-friendly, easily interpretable, sensitive and specific (to avoid false negative and positive results). Another aspect that should always be considered in the development of point of care diagnostic devices is their size. They need to be transportable and in the best case being pocket size. Furthermore, such devices need to be affordable, although the threshold for the market price is different depending on the socio-economic environment of the patient(s). In particular, one of the technical developments of the past decade is in favor of such developments: the smartphones (and tablets) are ever improving mini-computers with innovative interaction interfaces that often “only” require particular sensors and the complementary software to turn into a diagnostic device. Even smartwatches (or fitness watches) already measure routinely heart rate and other health-related parameters start to become routine read-outs such as oxygen saturation (see, e.g., Garmin watch portfolio) or blood pressure. It is worthwhile to mention that most point of care devices work completely non-invasive or at least only require such small amounts of blood that can be taken by finger prick avoiding venous puncture (e.g., Pandey et al., 2018). Examples of early developments of smartphone-based diagnostic devices in conjunction with an “App” and appropriate sensors have been described for the detection of sickle cell disease (Knowlton et al., 2015; Ung et al., 2015), although not all point of care diagnostic devices are smartphone based. Promising recent developments are the HemoTypeSC to determine the hemoglobin types by Silver Lake Research (Azusa, CA, United States) (e.g., Nankanja et al., 2019; Steele et al., 2019; Mukherjee et al., 2020) or Sickle SCAN with a similar application by BioMedomics (Morrisville, NC, United States) (Nguyen-Khoa et al., 2018). Also, a recent enrichment on the market was Q-POC by QuantuMDx (Newcastle, United Kingdom). Although initially developed to diagnose infectious diseases, it is also tested to diagnose all the different β-thalassemia mutations (Elion, 2019).

A very special test for sickle cell disease was recently developed. The test is utilizing filter paper and, thus, costs less than 0.05€ per test (Delobel et al., 2018).

## A New Generation of Devices Probing for Physico-Chemical Properties of Red Blood Cells

As outlined in the introduction, although the identification of a molecular lesion is mandatory in genetic disorders to confirm the diagnosis, clinical observations reveal that the genotype/phenotype correlation is not always possible and that other genetic/epigenetic factors (other than the molecular defect) may contribute to the clinical phenotype. This is particularly evident in case of intrafamily clinical variability in presence of the same mutation, or in clinical variability in the same patient during his life (Grace et al., 2018; Bianchi et al., 2020). Therefore, diagnostic devices that address physico-chemical properties such as cellular deformability, hemolysis, and red blood cell interaction properties entered the market or are under development.

**FIGURE 1:**
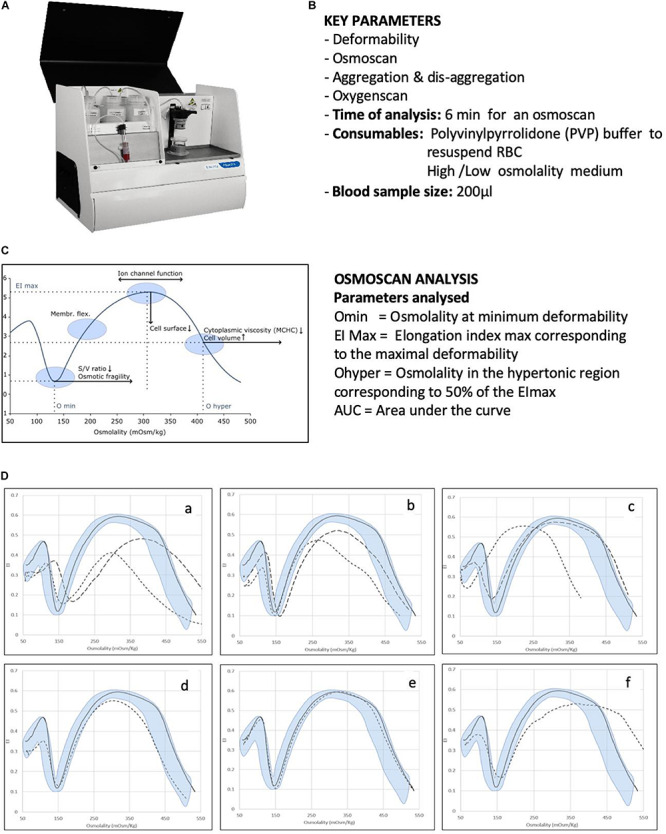
Analysis of RBC membrane disorders and other rare haemolytic anaemias by ektacytometry analysis. **(A)** Image of the Laser Optical Rotational Red Cell Analyzer (LoRRca Maxsis RR Mechatronics, Netherlands). **(B)** List of key parameters analyzed by the instruments. **(C)** Osmoscan profile in normal subjects and parameters analyzed: the Omin value represents the 50% of the RBCs hemolysis in conventional osmotic fragility assays, reflecting mean cellular surface-to volume ratio; the Elongation Index (EI) max corresponds to the maximal deformability obtained near the isotonic osmolality and is an expression of the membrane surface; the Ohyper reflects mean cellular hydration status; the AUC correspond to the area under the curve beginning from a starting point in the hypo-osmolar region and an ending point in the hyper-osmolar region. **(D)** Examples of typical osmoscan profiles in hemolytic anemias resulting from the analysis of 202 patients affected by congenital hemolytic anemia of different etiology. Continuous line represents a daily control and shaded area the control range curve. **(a)** HS = hereditary spherocytosis, **(b)** HE = hereditary elliptocytosis, **(c)** HSt = hereditary stomatocytosis: HSt-PIEZO1 (hereditary xerocytosis) (dotted line), HSt-KCNN4 (Gardos channelopathy) (dashed line), **(d)** CDAII = congenital diserythropoietic anemia type II, **(e)** RBC enzymopathies (pyruvate kinase deficiency), **(f)** other rarer RBC enzymopathies (glucosephosphate isomerase deficiency). Panels **(A)** and **(C)** are reproduced with permission from RR Mechatronics. Panels **(D)** is reproduced from Zaninoni et al. (2018).

An example addressing cell deformability is the LoRRca (RR Mechatronics, Hoorn, Netherlands) (Figure 1). The instrument was developed and commercialized only some years ago. Initially, it was used in a few highly specialized centers, but now, it is increasingly used to routinely diagnose rare red cell disorders (Da Costa et al., 2016; Zaninoni et al., 2018). Moreover, its new oxygen scan application, measuring the relative oxygen pressure at the critical point the red blood cells start to sickle, might offer in the future new opportunities for monitoring sickling during new treatment strategies, personalized medicine, and prediction of complication in sickle cell disease (Rab et al., 2020).

A neat concept to test red blood cell stability based on a bead mill and spectral measurement of hemoglobins was developed by Blaze Medical Devices (Ann Arbor, MI, United States). Although the concept was convincing (Tarasev et al., 2016), the device never entered the market. However, measurements as a service are offered by Functional Fluidics (Detroit, MI, United States).

Yet another example is a table top device called MeCheM (mechanical and chemical modulator) that was developed by Epigem Ltd. (Redcar, United Kingdom) within the project CoMMiTMenT (Combined Molecular Microscopy for Therapy and Personalized Medication in Rare Anemias Treatments, funded in the European Community 7th Framework Program). It is a microfluidic device that can challenge red blood cells chemically or with functional surfaces, while red blood cells are microscopically observed. Although it is not yet on the market, it is under investigation in several hematologic laboratories within Europe and in a clinical trial testing the efficiency of Memantine for the treatment of sickle cell disease (Makhro et al., 2020).

## Novel Screens Based on High-Tech, Robotics, and Artificial Intelligence

**FIGURE 2:**
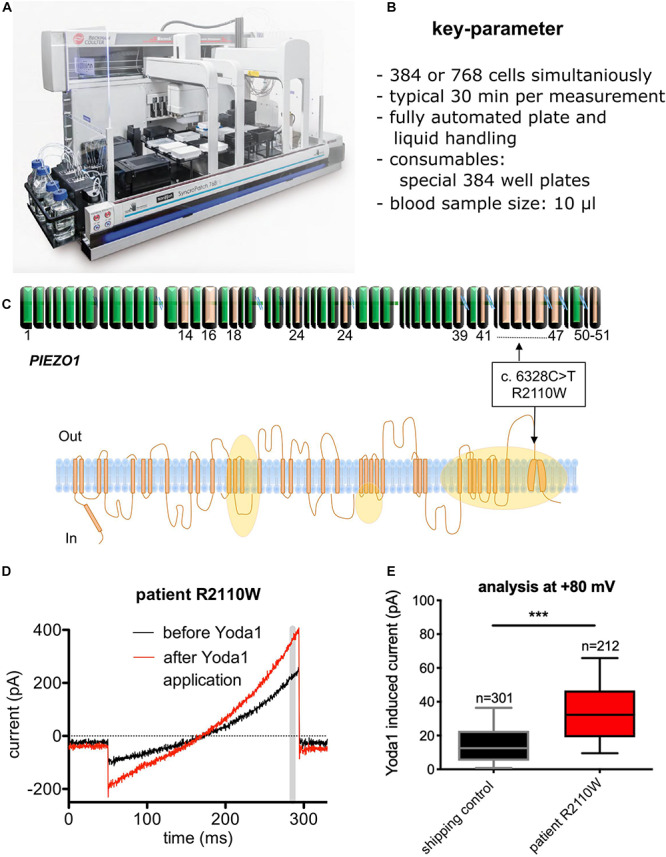
Diagnosis of a novel *PIEZO* mutation with automated patch-clamp technology. **(A)** Image of the SyncroPatch device (Nanion Technologies, Munich, Germany). **(B)** List of key parameters of the SyncroPatch. **(C)** Illustration of a novel mutation (R2110W) of the Piezo 1 ion channel. Although detected *per se*, it was unknown if the mutation has a functional effect on the red blood cells. Orange areas represent regions affected by previously reported mutations. **(D)** Raw data traces of a red blood cell recording for illustration. Yoda1 is a specific activator of Piezo 1. The gray bar depicts the time point (= membrane potential), which was used for the statistical analysis. **(E)** Statistical analysis of all measured cells (R2110W mutation vs. control) to exemplify the functional impact of the mutation. n gives the number of successful measured and analyzed cells. **(A)** Reproduced with permission from Nanion Technologies. **(C–E)** Reproduced from Rotordam et al. (2019) with permission of the Ferrata Storti Foundation.

The opposite of point of care devices are machines or procedures that are so complicated and/or expensive that they are exclusively established in expert centers or even in specialized research laboratories. For these devices/procedures, it is sometimes hard to distinguish between the generation of new knowledge and diagnosis—at least this borderline is fuzzy. An example of such a device is an automated patch-clamp robot that in the past proofed to be useful for the investigation of red blood cells (Makhro et al., 2013; Minetti et al., 2013). An image of the device and the joint test is given in Figure 2. This patch-clamp robot, originally developed for pharmacological compound screening, was used for a functional diagnosis of a new variant of a mutation of the mechanosensitive ion-channel Piezo1, which is associated with hereditary xerocytosis (Rotordam et al., 2019).

Another procedure used to understand/diagnose diseases is the *in vitro* erythropoiesis, which was refined and optimized considerably also in the past decade. Although bioreactors for an automated and controlled differentiation from peripheral stem cells to erythrocytes are under development (Heshusius et al., 2020), up to date, *in vitro* erythropoiesis still requires the human resource of a scientist or technician to be performed. However, we like to emphasize that recent results showed the importance of erythropoiesis for determination of the severity of the disease (Moura et al., 2019; Caulier et al., 2020).

Artificial intelligence based on artificial neuronal networks is a concept that will enter all incarnations of diagnostic devices as long as they involve computational power. A very old and established diagnostic tool, the analysis of blood smears, is currently reinvented based on the recordings of confocal stacks, three-dimensional rendering of the cells, and classification of the cell shapes by artificial neural networks (compare Bogdanova et al., 2020 within this research topic). Whether it will indeed be possible to link the occurrence of particular cell shapes with concrete mutations still needs to be explored.

## Logistic Concepts for High-Tech Diagnosis and Research

Given the rarity and the heterogeneity of this group of disorders, the confinement between research and diagnostics is sometimes faint, especially for the rare or undiagnosed diseases. In the presence of very rare disorders (e.g., some rare red blood cell enzyme defects or in defects of cell volume regulation), each case seems unique and worth to be described and deeply characterized. Networking activities to recruit similar cases and to joint expertise and collaborations is utmost important in this field (e.g., Vives Corrons et al., 2014; Fermo et al., 2017; Petkova-Kirova et al., 2019). However, this requires a logistic organization of collaboration, in particular for sharing the blood samples. The most common mode is the shipment of samples. This is easy and straightforward when cells or cell extracts can be preserved, like for blood smears, chemically fixed cells for morphological investigations (Abay et al., 2019), isolated RNA for genetic investigations, frozen cells, e.g., for protein analysis, etc. However, it is much more complicated when assays are based on living cells. Some years ago, we performed a dedicated study on healthy red blood cells to mimic transportation conditions (Makhro et al., 2016). The outcome was surprising in the respect that different red blood cell parameters require different conditions in terms of anticoagulant and temperature to resemble the results of fresh red blood cells. With cells of patients, the situation can be even worse. In a recent study on cellular intracellular Ca^2+^ (Hertz et al., 2017), the effect of the transportation was bigger than the effect of the disease. In this particular study, the data could be “rescued” by normalizing to healthy transportation controls. However, we also found that in certain conditions, differences between patients and control can easily be lost during some hours *in vitro* (Rotordam et al., 2019). Taking all these indications and although shipment of blood samples is the most common and popular method of interlaboratory collaboration, it is by far not an ideal configuration. However, a much better option would be if the patients travel to the specialized laboratories. Although we recently introduced this practice in our laboratory, it does work only for a minority of patients (due to the state of the patients, their compliance, or other restrictions) and is only a kind of control that hardly can reach statistical power, especially for rare and very rare diseases. The third option would be mobile specialized laboratories. Surprisingly, this idea, so far, got stuck within discussion among researchers, presumably because appropriate funding programs are the restriction. From the technical point of view, (i) we are, in principle, able to catapult even confocal microscopes with biological samples into space (Thiel et al., 2019) and (ii) an increasing number of devices are designed for transportation. This is not restricted to the classical point of care devices mentioned above but also applies for fairly complicated machines such as flow cytometers (e.g., CyFlow Cube6, Sysmex, Germany). Therefore, a mobile laboratory down on earth for red blood cell-related diseases should be a challenging project but with high chances of success and only limited risks as it was realized for other purposes before (e.g., Weidmann et al., 2018). However, the intended project/use should define whether such a laboratory should be on wheels, on a boat, or on board an aircraft.

## Conclusion and Outlook

Point of care, artificial intelligence, and personalized theranostocs are probably the major keywords that characterize current and future developments in diagnostic devices for red blood cell-related diseases, in general, and for rare anemias, in particular. From a conceptual point of view, genetic analysis is well established, and decrease in the size of devices and significant cost drops will increase the spread and the regular use of this diagnostic tool. In line with this, a targeted analysis of a defined (group of) protein(s) will be replaced by a full genome analysis. However, functional analysis (more and more based on individual cells) on top of gene characterization will become increasingly important. This may go far beyond the samples given above and is likely to include further miniaturized assays of well-known tests such as the investigation of cell density distributions or measurements considering filterability properties.

## Author Contributions

Both authors wrote and approved the manuscript.

## Conflict of Interest

The authors declare that the research was conducted in the absence of any commercial or financial relationships that could be construed as a potential conflict of interest.
